# A microglial compliment: controlling neuronal function from within

**DOI:** 10.1038/s41392-024-01989-9

**Published:** 2024-10-07

**Authors:** Dilara Hasavci, Thomas Blank

**Affiliations:** Institute of Neuropathology, Faculty of Medicine, University of Freiburg, Freiburg, Germany

**Keywords:** Cellular neuroscience, Neuroimmunology, Ageing

A recent study published in *Cell* revealed that in the aging brain, the microglia-derived complement component C1q is internalized into neurons through endocytosis, integrates into ribonucleoprotein (RNP) complexes where it inhibits neuronal protein synthesis and alters the protein content. These findings demonstrate an unexpected intracellular function of C1q in neurons with significant implications for understanding age-related changes in brain function and potentially neurodegenerative diseases.^1^

**Fig. 1:**
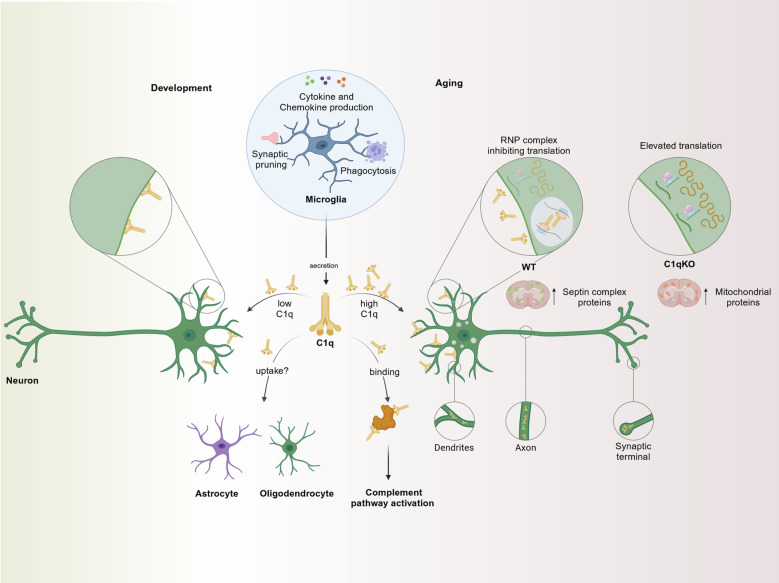
Novel function of microglia-derived complement protein C1q during aging. Microglia play a central role in immune responses, phagocytosis of cellular debris and synaptic pruning during early postnatal stages. C1q is produced by microglia during development and aging. It can bind to its receptor on neurons, where it mediates development-associated synaptic pruning. During aging, however, C1q is present at higher levels and is taken up by neurons via endocytosis, where C1q accumulates in synaptic terminals, axons and dendrites. C1q is integrated into the ribonucleoprotein (RNP) complex, where it inhibits protein homeostasis. Proteins associated with septin complexes are enhanced in WT brain tissue of aged mice while mitochondrial proteins are enriched in brain tissue of aged C1q-deficient mice. C1q might also be taken up by astrocytes or oligodendrocytes and activate the classical complement pathway after binding to misfolded proteins or cellular debris. This figure was created with BioRender.com

Microglia are responsible for surveilling the central nervous system, responding to changes and injuries, phagocytosing cellular debris and protein aggregates, pruning synapses during development, releasing factors that influence neural function, and mediating immune responses in the brain^2^ (Fig. 1). The current study adds another facet to our understanding of microglial function by demonstrating the inhibitory effects of C1q on neuronal protein homeostasis during aging. The researchers discovered that C1q interacts with neuronal RNP complexes in an age-dependent manner (Fig. 1). Using advanced biochemical and imaging techniques, they demonstrated that purified C1q undergoes RNA-dependent liquid-liquid phase separation (LLPS) in vitro. This phase separation is crucial for C1q’s interaction with neuronal RNP complexes in vivo, which is dependent on RNA and endocytosis.^1^ The study identified that the collagen-like domain of C1q is critical for this neuronal uptake. Once inside neurons, C1q binds to ribosomal proteins, RNA-binding proteins and RNA, impairing neuronal protein synthesis and homeostasis.^1^ Even though C1q has no effect on neuronal protein translation in young adult (2-3 months) animals, deletion of C1q in adult animals (1 year) resulted in a significant increase in protein translation. Global proteomic analysis further demonstrated brain-wide changes in protein content between 1-year-old WT and C1q-deficient littermates, revealing an unexpected enrichment of proteins associated with septin complexes in adult WT brain tissue and mitochondrial proteins in adult C1q-deficient brain tissue.

Scott-Hewitt et al. also explored the functional consequences of C1q integration into neuronal RNP complexes. Young adult mice deficient in C1q displayed impaired fear memory extinction, indicating that C1q is essential for certain cognitive functions.^1^

This groundbreaking study by Scott-Hewitt et al.^1^ adds another layer on the highly age-dependent role of C1q in brain function. The authors were able to show that C1q levels in the brain rise dramatically with age, suggesting an involvement in age-related impairment of brain function.^1^ In line with this assumption, a previous study showed that aged C1q-deficient mice (17 months) exhibited considerably less cognitive and memory deterioration.^3^ This study by Stephan et al. found that C1q-deficiency selectively protects from aging-related cognitive decline when analyzing spatial learning and memory, including reversal learning in the Morris Water Maze and spatial working memory (Y-Maze).^3^

Notably, the authors did not detect differences between C1q-deficient mice and their wild-type littermates in any of the behavioral tasks performed when animals were 3-month-old adults.^3^

Scott-Hewitt et al. described in 2-3-month-old mice impaired cognitive function in the absence of C1q for a very specific form of learning, namely fear extinction learning while fear memory acquisition or retrieval were normal. An important inquiry emerges regarding the specific contributors to this observed cognitive limitation. Despite fear extinction’s reliance on neuronal protein synthesis, young adult wild-type and C1q-deficient mice showed no significant differences in this process. Although the overall rate of protein synthesis in neurons might still be comparable in both groups, there might be differences in protein content between young adult wild-type and C1q-deficient mice. This scenario could result in C1q-deficient mice having lower levels or a complete absence of proteins essential for fear extinction learning.

Even though the exact mechanism remains unclear, the presence of C1q is required for certain aspects of learning and memory in young adult mice, as demonstrated in this instance of decreased fear memory extinction displayed by C1q-deficient mice.^1^ In contrast, age-related elevation of C1q protein levels has a negative impact on cognitive performance, especially regarding hippocampus-dependent learning tasks.^3^

From this outlined data, it is tempting to speculate that other cell types in the brain, like astrocytes or oligodendrocytes may also take up C1q under certain conditions like increasing age. Along these lines, one might wonder how different cell types internalize C1q and what might be the cell type-specific functional implications under physiological and pathological conditions (Fig. 1)? Even under normal conditions, a continuous low-level C1q-release seems to be present. C1q might play a role in maintaining the stability of RNPs, potentially helping to preserve their structure and prevent them from breaking apart. This stabilizing effect could be one way that C1q influences protein balance in neurons, possibly by controlling protein production in specific areas of the cell (Fig. 1). While Scott-Hewitt et al. focused mainly on the developmental and aging aspects, subsequent studies might elucidate whether additional stimuli like injury to the CNS or peripheral inflammatory conditions also initiate enhanced C1q uptake and intracellular accumulation in cells of the brain.

By illuminating the role of C1q in neuronal protein homeostasis and cognitive function, the research opens new avenues for exploring therapeutic strategies targeting C1q and its interactions in the brain. Reducing C1q levels or its integration into neuronal RNP complexes could potentially mitigate age-related cognitive decline and neurodegenerative conditions, where protein misfolding and aggregation are widespread. Binding of C1q to misfolded proteins or cellular debris can activate the classical complement pathway, potentially contributing to inflammation in neurodegenerative diseases.^4^ Another unexpected finding of this study was the enrichment of mitochondrial proteins in brain tissue from adult C1q-deficient mice.^1^ It is conceivable that the age-related increase in C1q proteins leads to mitochondrial damage and dysfunction, which facilitates the accumulation of misfolded proteins and decreased energy production triggering a weakening of cognitive capabilities.

Given the critical function of C1q in fear extinction, possible treatment methods targeting C1q should not be limited to age-related cognitive decline but can also be extended to patients with neuropsychiatric disorders (Fig. 1). As an example, persistent fear memories, frequently observed in anxiety and trauma-related disorders,^5^ can hinder the success of exposure therapies that depend on functional and effective fear extinction mechanisms.
